# A Rare Presentation of Posterior Reversible Encephalopathy Syndrome in a Previously Healthy Young Male: A Case Report

**DOI:** 10.7759/cureus.92870

**Published:** 2025-09-21

**Authors:** Jatin Goyal, Morgan McMasters, Matthew Jordan, Rushil Randive

**Affiliations:** 1 Surgery, Florida International University, Herbert Wertheim College of Medicine, Miami, USA; 2 Anesthesiology, Florida International University, Herbert Wertheim College of Medicine, Miami, USA; 3 Neurosurgery, Florida International University, Herbert Wertheim College of Medicine, Miami, USA; 4 Critical Care, Baptist Health South Florida, Miami, USA

**Keywords:** acute hydrocephalus, cerebellar edema, hypertensive crisis, posterior reversible encephalopathy syndrome (pres), reversible posterior leukoencephalopathy syndrome (rpls), vasogenic brain edema

## Abstract

Posterior reversible encephalopathy syndrome (PRES) is a neurological disorder characterized by vasogenic cerebral edema, typically associated with hypertension, renal failure, or immunosuppressive therapy. We present a unique case of a 19-year-old, previously healthy male who developed PRES in the setting of an acute hypertensive crisis, with an atypical presentation including severe cerebellar edema and obstructive hydrocephalus, necessitating emergent neurosurgical intervention. Unlike typical cases, this patient had no prior history of hypertension or known risk factors, highlighting the importance of considering secondary causes of hypertension in young individuals. His hospital course was further complicated by visual impairment due to hypertensive retinopathy and pneumonia, requiring bilevel positive airway pressure (BiPAP) support. This case expands the spectrum of PRES presentations, emphasizing that acute, severe hypertension alone can precipitate life-threatening complications. Early recognition, aggressive blood pressure management, and thorough evaluation for underlying etiologies are crucial in optimizing patient outcomes.

## Introduction

Posterior reversible encephalopathy syndrome (PRES), also known as reversible posterior leukoencephalopathy syndrome (RPLS), is a clinical and radiologic diagnosis of white matter damage, which generally occurs in the posterior lobes of the brain in the setting of vasogenic edema and capillary leak [1]. PRES rarely involves the cerebellum. It most commonly presents with headache, visual disturbances, altered mental status, and seizures [2]. Therefore, the initial differential diagnosis is broad, as it overlaps with the clinical pictures of primary meningitis, substance abuse or intoxication, acute ischemic stroke, cerebral vasculitis, and neoplastic processes. 

The pathogenesis of PRES is thought to involve abrupt increases in blood pressure, which result in disordered cerebral autoregulation, leading to endothelial dysfunction, vasogenic edema, and ischemia. Known risk factors include immunosuppressive medications, immunocompromised disease states, pregnancy, renal disease, idiopathic hypertension, hemoglobinopathies, and autoimmune disease. While this syndrome more commonly affects adult patients, PRES has also been reported in pediatric and adolescent populations with prior exposure to immunosuppressive therapies or underlying immune system dysfunction [3]. 

In this report, we present a unique case of PRES in a 19-year-old male with no significant past medical history, who developed involvement of the posterior and cerebellar circulation in the setting of acute-onset, profound hypertension.

## Case presentation

A 19-year-old, previously healthy Guatemalan male presented to the Emergency Department with a severe headache and abdominal pain that began earlier in the day, preventing him from attending work. His father had found him in bed with facial trauma, which the patient stated was due to a fall at home, though he did not recall the rest of the events leading up to his arrival at the Emergency Department. He appeared intoxicated and unsteady, although he denied any alcohol consumption. On initial presentation, he was found to have severe hypertension (233/155 mmHg) and tachycardia, requiring the initiation of a nicardipine drip. In addition to profound hypertension, his neurologic exam was only remarkable for poor language fluency, limited to “yes” and “no” responses, with a Glasgow Coma Scale (GCS) of 14. Cranial nerves II-XII were grossly intact, pupils were equal and reactive, and motor strength was 5/5 throughout, with no pronator drift. Sensory examination was intact to light touch and pinprick. Coordination and gait could not be reliably assessed. The remainder of the physical exam was unremarkable. Initial laboratory results showed leukocytosis (12.12 K/µL) and kidney injury (creatinine: 1.32 mg/dL, estimated glomerular filtration rate (eGFR): 80) (Table 1).

**Table 1:** Key laboratory results over the course of hospitalization Abbreviations: WBC: White Blood Cells; eGFR: estimated Glomerular Filtration Rate

Labs	Day 1	Day 2	Day 3	Day 4	Day 5	Day 6	Day 7	Day 8	Day 9	Day 10	Day 11	Day 12	Day 13	Day 14	Day 15	Day 16	Day 17	Day 18	Day 19	Day 20	Day 21	Day 22	Reference Range
WBC (K/µL)	12.12	8.77	9.71	15.98	9.95	7.22	8.89	8.76	9.31	9.01	9.62	8.28	11.75	12.69	10.57	13.28	12.85	14.13	8.61	8.28	7.64	7.37	3.40-11.00
Creatinine (mg/dL)	1.32	1.18	1.35	1.42	1.46	1.04	1.35	1.34	1.49	1.68	1.75	1.46	1.78	1.51	1.63	1.35	1.63	1.38	1.49	1.3	1.48	1.53	0.70-1.30
eGFR	80	>90	78	71	>90	>90	78	78	69	60	57	71	56	68	62	78	62	76	69	81	69	67	>90

**Figure 1:**
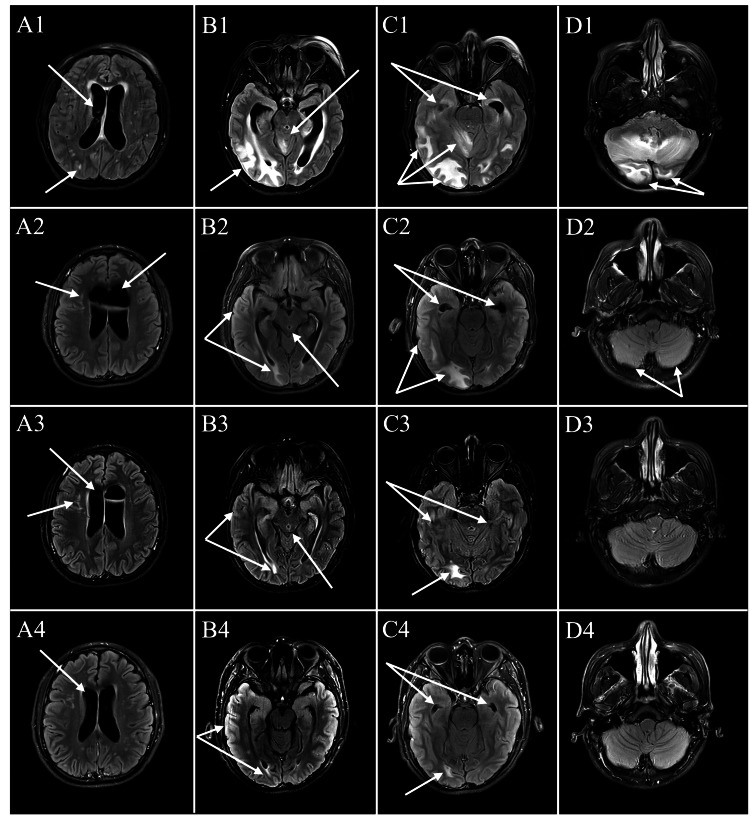
Serial FLAIR MRI during hospitalization Day 1 (A1-D1): Hydrocephalus with subependymal edema, scattered white matter hyperintensities, and hyperintensity involving the right occipital/temporal lobes and bilateral cerebellum. Temporal horns are present, consistent with hydrocephalus. Day 4 (A2-D2): Partial improvement with reduced subependymal edema, fewer white matter lesions, and decreased hyperintensity in the right occipital/temporal lobes and cerebellum. Day 10 (A3-D3): Continued improvement with diminished hydrocephalus, near-resolution of white matter lesions, and reduced signal abnormality in the right occipital lobe. Cerebellar findings are stable. Day 17 (A4-D4): Marked improvement with minimal residual subependymal edema, resolution of occipital/temporal involvement, and stable cerebellar findings. Abbreviation: FLAIR MRI: Fluid-Attenuated Inversion Recovery Magnetic Resonance Imaging

Upon admission to the hospital, the patient was immediately placed in the intensive care unit (ICU) due to concern regarding the risk of cardiopulmonary arrest from brainstem compression. Initial brain magnetic resonance imaging (MRI) without contrast revealed extensive vasogenic cerebral edema, particularly in the posterior fossa, with mass effect on the fourth ventricle and crowding of the foramen magnum, with associated obstructive hydrocephalus (Figure 1). Cerebrospinal fluid (CSF) analysis was mostly benign, showing lymphocytes, monocytes, rare neutrophils, and a few red blood cells (Table 2). Leading differentials at this time included central nervous system vasculitis and metastatic disease. Neurosurgery was consulted for an emergent right frontal ventriculostomy placement to address the hydrocephalus, and levetiracetam and dexamethasone were initiated.

**Table 2:** Cerebral spinal fluid laboratory results Abbreviations: CSF: Cerebral Spinal Fluid; PCR: Polymerase Chain Reaction

CSF Laboratory Values	Results	Reference Range
Color	No color	No color
Appearance	Clear	Clear
Total nucleated cells (cells/µL)	8	0-5
Red blood cell count (cells/µL)	24	0
Neutrophils (%)	25	<5
Lymphocytes (%)	41	60-80
Monocytes (%)	34	15-45
Protein (mg/dL)	38	<45
Glucose (mg/dL)	94	50-80
Xanthochromia	Absent	Absent
Toxoplasmosis	Negative	Negative
*Escherichia coli* by K1 PCR	Not detected	Not detected
*Haemophilus influenzae* PCR	Not detected	Not detected
*Listeria monocytogenes* PCR	Not detected	Not detected
*Neisseria meningitidis* PCR	Not detected	Not detected
*Streptococcus agalactiae* PCR	Not detected	Not detected
*Streptococcus pneumoniae* PCR	Not detected	Not detected
Cytomegalovirus PCR	Not detected	Not detected
Herpes simplex virus 1 PCR	Not detected	Not detected
Herpes simplex virus 2 PCR	Not detected	Not detected
Human herpesvirus 6 PCR	Not detected	Not detected
Human parechovirus PCR	Not detected	Not detected
Varicella zoster virus PCR	Not detected	Not detected
*Cryptococcus neoformans*/*gattii* PCR	Not detected	Not detected

Given the concern for potential infectious causes, empirical antibiotic therapy with ceftriaxone and vancomycin was initiated, and the patient was later started on albendazole and metronidazole, after a positive toxoplasmosis IgG serology.

**Figure 2:**
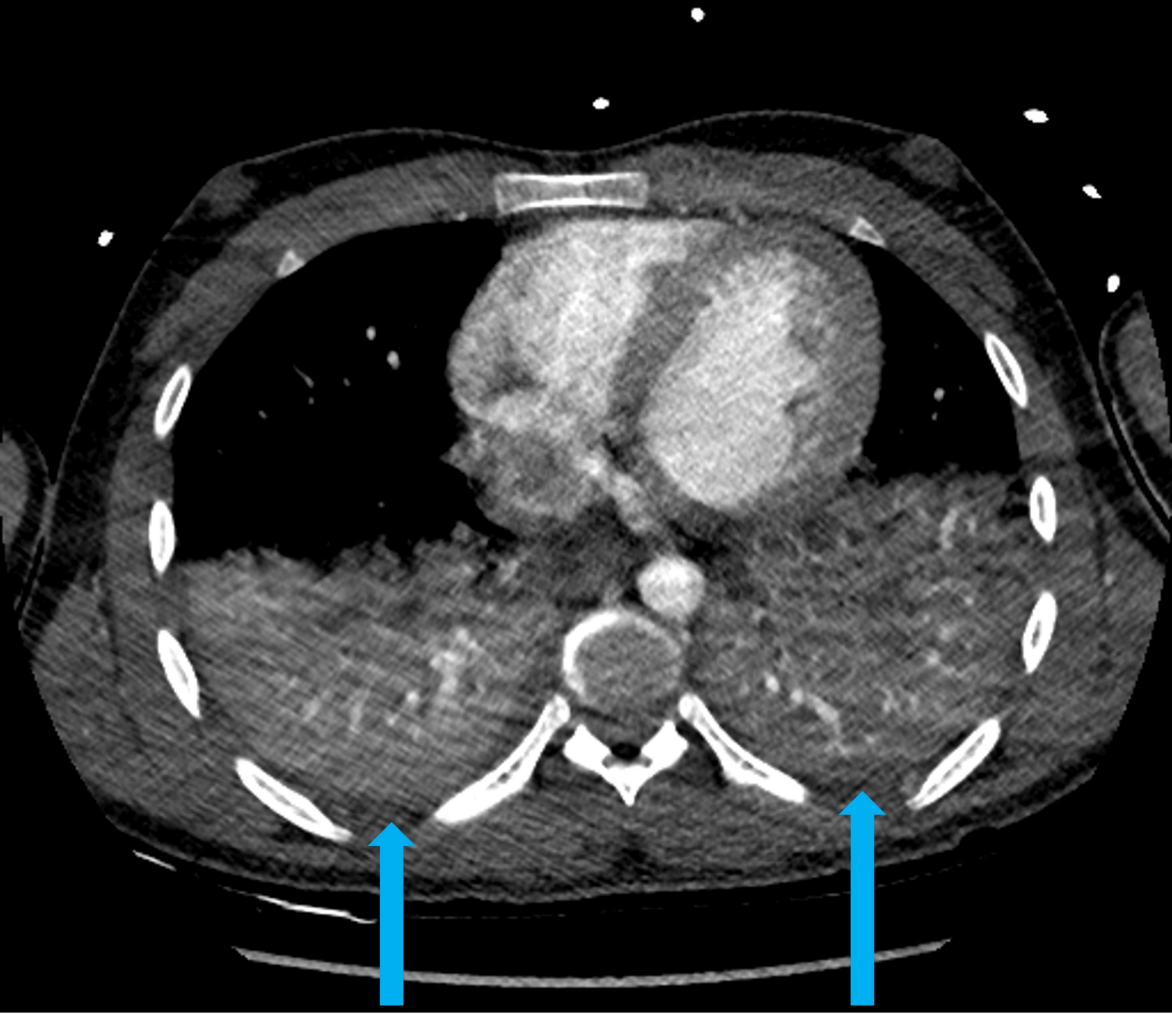
Chest computed tomography from hospitalization day 4 Blue arrows indicate large bilateral lower lobe consolidations.

Additionally, a chest computed tomography (CT) scan on day 4 of admission had revealed large bilateral lower lobe consolidations concerning for pneumonia (Figure 2), requiring bilevel positive airway pressure (BiPAP) support and a seven-day course of piperacillin-tazobactam therapy. Follow-up brain MRI without contrast on day 4 showed improving cerebral edema (Figure 1). A diagnostic cerebral angiogram on day 8 revealed no evidence of vasculitis or other vascular abnormalities. By day 10, a repeat brain MRI without contrast demonstrated stable ventriculomegaly, resolving cerebellar edema, and persistent scattered T2 fluid-attenuated inversion recovery (FLAIR) hyperintensities (Figure 1). Per the neurology team, the patient’s clinical improvement effectively ruled out a neoplastic etiology.

**Figure 3:**
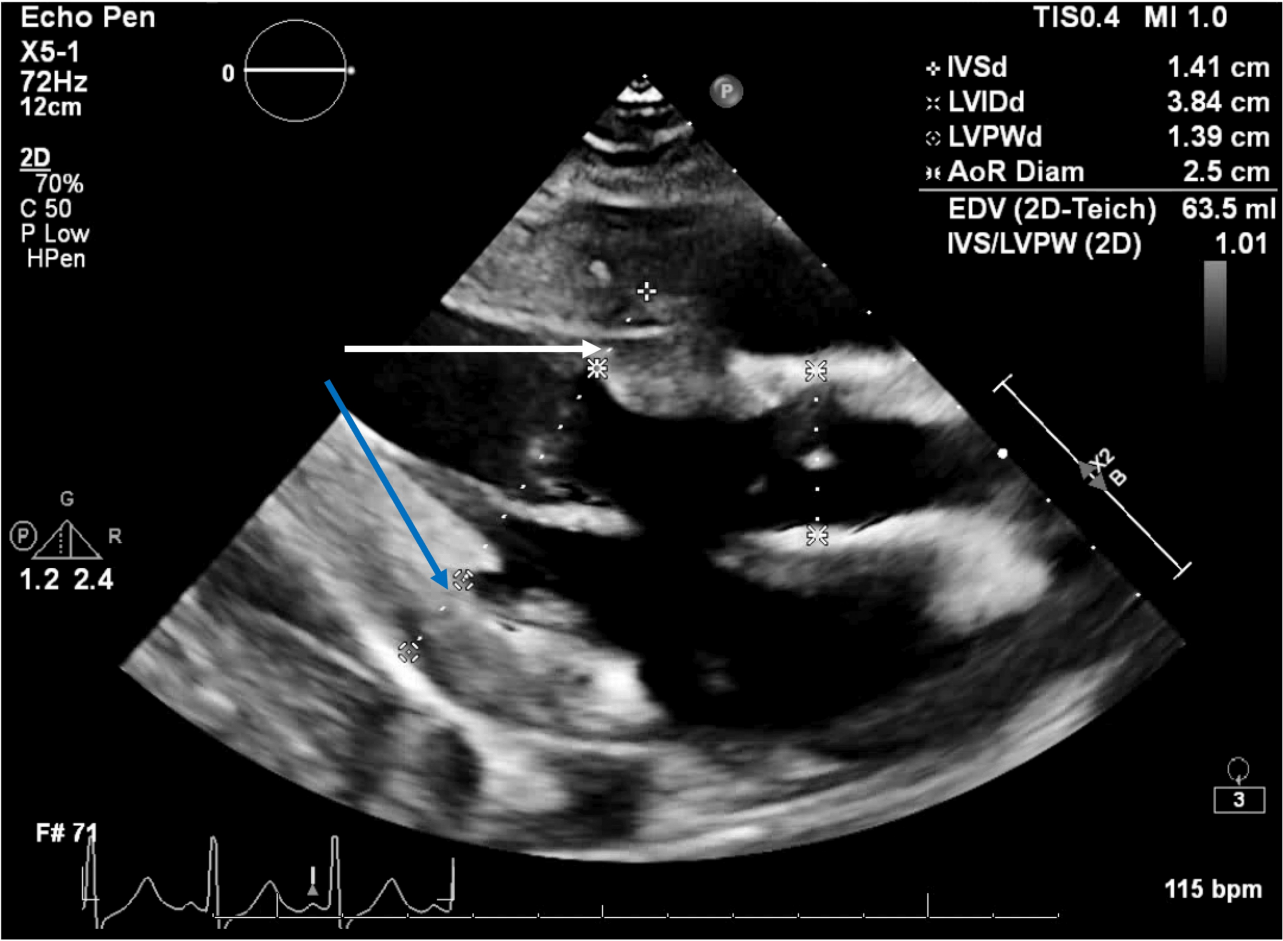
Echocardiogram indicating left ventricular hypertrophy The patient’s mildly elevated IVSd (1.41 cm; normal ≤ 1.0 cm, white arrow) and LVPWd (1.38 cm; normal ≤ 1.0 cm, blue arrow) indicate mild left ventricular hypertrophy. Measurements are indicated by calipers and corresponding values in the upper right corner. Abbreviations: IVSd: Interventricular Septal Thickness in Diastole; LVPWd: Left Ventricular Posterior Wall Thickness in Diastole

The patient’s hospital course was further complicated by abnormalities in multiple organ systems. Ophthalmologic examination showed acutely worsened vision, consistent with blindness in bilateral visual fields, determined to be due to involvement of the occipital region of the brain and not direct pathology of the eyes. Regarding the cardiovascular system, persistent hypertension required treatment with nifedipine, and later transitioning to losartan. Echocardiography showed mild to moderate left ventricular hypertrophy, with a normal ejection fraction of 55%-60% (Figure 3). A negative QuantiFERON test ruled out tuberculosis.

By day 15, the patient's external ventricular drain was clamped. Otolaryngology was consulted for epistaxis, which was attributed to dry nasal mucosa and managed with mupirocin. On hospital day 20, the external ventricular drain was removed, and the patient was downgraded from the ICU. On the patient’s last day in the hospital (day 22), his blood pressure was still elevated (136/98 mmHg), which required an increase in losartan dosing. His follow-up lab work showed resolution of leukocytosis, with continued mild kidney injury (creatinine: 1.53 mg/dL, eGFR: 67) (Table 1). Throughout his hospitalization, his laboratory values fluctuated continuously, and key parameters - such as white blood cell count and eGFR - were closely monitored not only to guide his clinical management, but also to monitor for the emergence of new or nosocomial complications. Given the significant clinical and radiologic improvement, the patient was discharged with instructions to follow up on his persisting symptoms and final diagnosis of PRES as an outpatient. At discharge, he had returned to baseline functional status, with no residual neurological deficits aside from mild visual impairment, which ophthalmologic evaluation attributed to hypertensive retinopathy.

## Discussion

PRES is a neurological disorder characterized by vasogenic cerebral edema, commonly associated with hypertension, renal failure, eclampsia, autoimmune disease, or immunosuppressive therapy [4]. The pathophysiology of PRES is thought to involve a failure of cerebral autoregulation, leading to hyperperfusion, endothelial dysfunction, and subsequent blood-brain barrier disruption. Typical radiologic findings include symmetric white matter edema, particularly in the parieto-occipital regions [5]. However, atypical presentations - including cerebellar involvement and hydrocephalus - have been reported but remain uncommon.

This case presents a unique manifestation of PRES in a previously healthy 19-year-old male without a known history of hypertension. The patient’s presentation with severe headache and altered mental status, along with imaging findings of extensive vasogenic cerebral edema, cerebellar swelling, and hydrocephalus, adds to the growing recognition of atypical features in PRES. Unlike the classic presentation involving posterior cerebral regions, this case featured significant cerebellar involvement, causing brainstem compression and obstructive hydrocephalus, requiring emergent neurosurgical intervention. Cerebellar edema, although rare in PRES [6-8], has been linked to severe outcomes such as hydrocephalus, brainstem compression, and the need for external ventricular drainage. The pathophysiological mechanism of cerebellar involvement remains unclear but may involve greater disruption of cerebellar autoregulation or differential vulnerability of the posterior circulation.

Beyond neuroimaging findings, this case also highlights the role of social and structural determinants of health in diagnosis and care. The patient's social history was notable for recent immigration to the United States from Guatemala, with poor prior medical follow-up. Although there were no known chronic medical conditions, an underlying illness predisposing to hypertensive emergency may have been present but undiagnosed, owing to limited healthcare access. The patient’s persistently reduced eGFR, despite blood pressure control, raises concern for previously undiagnosed chronic kidney disease. In the context of limited prior medical follow-up and socioeconomic barriers, this case highlights how social history can directly contribute to delayed recognition of chronic hypertension or renal disease - conditions that may have increased vulnerability to hypertensive crisis and PRES.

In addition to these social factors, the patient underwent an extensive evaluation for secondary causes of hypertension, including renal artery Doppler imaging, thyroid function testing, plasma renin and aldosterone levels, and plasma/urine metanephrines. All results were negative, further supporting the idiopathic nature of his hypertensive crisis. Explicitly ruling out these etiologies strengthens the interpretation that acute, severe hypertension alone was the precipitating factor for PRES in this case.

Despite these negative findings, the patient’s altered mental status on arrival, which resembled intoxication, raised the possibility of drug-induced PRES, a known but often overlooked etiology. Toxic encephalopathies induced by medications, illicit drugs, or environmental toxins can mimic PRES both clinically and radiologically [9]. In this case, toxicology testing was negative, making drug-induced or toxic PRES less likely. While undocumented environmental or occupational exposures were considered as part of the differential, there was no supporting evidence, and these remain remote possibilities rather than probable contributors. Autoimmune disorders, such as systemic lupus erythematosus, and metabolic encephalopathies, including uremic and hepatic encephalopathy, were also considered due to their overlapping clinical and radiologic features [10].

Beyond these etiologies, clinicians must maintain a high index of suspicion and carefully distinguish them from other conditions with overlapping features. In this case, cerebral infarction was an early consideration, particularly posterior-circulation or watershed strokes, but MRI demonstrated vasogenic rather than cytotoxic edema, and no restricted diffusion was seen. Central nervous system infections, such as meningitis and encephalitis, were also considered; however, the absence of fever, CSF pleocytosis, or leptomeningeal enhancement made these unlikely. Demyelinating diseases, including acute disseminated encephalomyelitis, can produce multifocal white matter lesions, but these are typically asymmetric, often persistent, and not as responsive to blood pressure control. Other differential diagnoses included brain tumors and neoplasm-related encephalopathy, which usually present with focal mass lesions and contrast enhancement, and dural venous sinus thrombosis, which was excluded by negative vascular imaging. Autoimmune vasculitis was ruled out by normal angiography, while toxic and metabolic encephalopathies were considered but unsupported by laboratory or toxicology results. Finally, reversible cerebral vasoconstriction syndrome and mitochondrial disorders were considered, but the clinical course and imaging did not align with these diagnoses. Other mimickers, such as subarachnoid hemorrhage, seizure-related encephalopathy, and hypertensive encephalopathy, were also considered; however, the absence of hemorrhage, the lack of seizure-related imaging changes, and the resolution of edema with blood pressure control further supported PRES as the most consistent diagnosis [9,11-14].

PRES in young patients without a prior diagnosis of hypertension is rare. In this case, the extreme hypertensive crisis suggests a rapid, severe rise in blood pressure as the precipitating factor, rather than chronic hypertension. This highlights the importance of evaluating secondary causes of hypertension in young patients presenting with hypertensive emergencies. While an extensive infectious and autoimmune workup did not reveal a definitive etiology, the transient nature of the cerebral edema and its resolution with blood pressure control strongly supported the diagnosis of PRES. Early recognition and aggressive blood pressure management remain the mainstays of treatment [5], as timely intervention can lead to significant clinical and radiologic improvement, as observed in this patient.

Most documented cases of PRES occur in patients with chronic hypertension, renal disease, or immunosuppressive therapy [15]. Reports of PRES in young, previously normotensive individuals remain limited, particularly those progressing to complications like cerebellar edema and hydrocephalus [7,16-19]. Although this report is limited by the absence of outpatient follow-up, which prevents assessment of long-term outcomes, it nonetheless expands the understanding of PRES by emphasizing atypical neuroimaging features and the importance of identifying at-risk populations. It demonstrates that acute, severe hypertension, even in the absence of prior diagnoses, can precipitate life-threatening complications. This case also highlights the need for early recognition of cerebellar involvement, especially in cases complicated by hydrocephalus.

## Conclusions

This case highlights the importance of recognizing PRES as a potential complication of severe hypertension, even in young patients without a history of hypertension. Early diagnosis and aggressive blood pressure management were crucial in preventing further neurological deterioration and promoting recovery, highlighting the need for prompt intervention in such hypertensive emergencies. Given the atypical features in this case, including cerebellar involvement and hydrocephalus, this report emphasizes the necessity of considering a broader spectrum of presentations for PRES, especially in previously healthy individuals. Evidence-based recommendations include early identification of hypertensive crises and close monitoring for secondary causes of hypertension in young patients. This case contributes to the growing understanding of PRES and supports the need for further research into the pathophysiology of atypical presentations, the role of cerebellar edema, and optimal management strategies for severe complications like hydrocephalus and brainstem compression.

## References

[1] Hinchey J, Chaves C, Appignani B (1996). A reversible posterior leukoencephalopathy syndrome. N Engl J Med.

[2] Wang W, Zhao LR, Lin XQ, Feng F (2014). Reversible posterior leukoencephalopathy syndrome induced by bevacizumab plus chemotherapy in colorectal cancer. World J Gastroenterol.

[3] Hun M, Xie M, She Z (2021). Management and clinical outcome of posterior reversible encephalopathy syndrome in pediatric oncologic/hematologic diseases: a PRES subgroup analysis with a large sample size. Front Pediatr.

[4] Fugate JE, Claassen DO, Cloft HJ, Kallmes DF, Kozak OS, Rabinstein AA (2010). Posterior reversible encephalopathy syndrome: associated clinical and radiologic findings. Mayo Clin Proc.

[5] Triplett JD, Kutlubaev MA, Kermode AG, Hardy T (2022). Posterior reversible encephalopathy syndrome (PRES): diagnosis and management. Pract Neurol.

[6] Hage P, Kseib C, Hmaimess G, Jaoude PA, Noun P (2018). Recurrent posterior reversible encephalopathy syndrome with cerebellar involvement leading to acute hydrocephalus. Clin Neurol Neurosurg.

[7] Bălaşa R, Maier S, Baubec EG, Bajkó Z, Bălaşa A (2015). Cerebellar and brainstem variant of posterior reversible encephalopathy syndrome. Acta Neurol Belg.

[8] Grossbach AJ, Abel TJ, Hodis B, Wassef SN, Greenlee JD (2014). Hypertensive posterior reversible encephalopathy syndrome causing posterior fossa edema and hydrocephalus. J Clin Neurosci.

[9] Koksel Y, McKinney AM (2020). Potentially reversible and recognizable acute encephalopathic syndromes: disease categorization and MRI appearances. AJNR Am J Neuroradiol.

[10] Chaudhuri J, Basu S, Roy MK, Chakravarty A (2023). Posterior reversible leucoencephalopathy syndrome: case series, comments, and diagnostic dilemma. Curr Neurol Neurosci Rep.

[11] Cheng X, Li J, Lan Y, Liu J, Chen S, Lu G (2021). Cerebrovascular disease in the setting of posterior reversible encephalopathy syndrome. Front Neurol.

[12] Geocadin RG (2023). Posterior reversible encephalopathy syndrome. N Engl J Med.

[13] Pilato F, Distefano M, Calandrelli R (2020). Posterior reversible encephalopathy syndrome and reversible cerebral vasoconstriction syndrome: clinical and radiological considerations. Front Neurol.

[14] Tetsuka S, Ogawa T (2019). Posterior reversible encephalopathy syndrome: a review with emphasis on neuroimaging characteristics. J Neurol Sci.

[15] Shimizu Y, Tha KK, Iguchi A (2013). Isolated posterior fossa involvement in posterior reversible encephalopathy syndrome. Neuroradiol J.

[16] Li D, Lian L, Zhu S (2015). Isolated cerebellar involvement in posterior reversible encephalopathy syndrome. J Neurol Sci.

[17] Moon SN, Jeon SJ, Choi SS, Song CJ, Chung GH, Yu IK, Kim DH (2013). Can clinical and MRI findings predict the prognosis of variant and classical type of posterior reversible encephalopathy syndrome (PRES)?. Acta Radiol.

[18] Ettinger N, Pearson M, Lamb FS, Wellons JC 3rd (2014). Pediatric posterior reversible encephalopathy syndrome presenting with isolated cerebellar edema and obstructive hydrocephalus. J Neurosurg Pediatr.

[19] Wright JN, Shaw DW, Ishak G, Doherty D, Perez F (2020). Cerebellar watershed injury in children. AJNR Am J Neuroradiol.

